# Adherent vs. Free-Floating Neural Induction by Dual SMAD Inhibition for Neurosphere Cultures Derived from Human Induced Pluripotent Stem Cells

**DOI:** 10.3389/fcell.2018.00003

**Published:** 2018-02-06

**Authors:** Martje G. Pauly, Victor Krajka, Felix Stengel, Philip Seibler, Christine Klein, Philipp Capetian

**Affiliations:** Institute of Neurogenetics, University of Lübeck, Lübeck, Germany

**Keywords:** neural stem cell culture, induced pluripotent stem cells, neural induction, adherent neural induction, embryoid bodies, neural differentiation

## Abstract

Keeping neural stem cells under proliferation, followed by terminal differentiation, can substantially increase the number of neurons generated. With regard to the usability of proliferating neurospheres (NSPHs) cultures, adherent induction protocols have not yet been studied in comparison to embryoid body (EB)-based protocols. To compare these proctocols, neural induction of human induced pluripotent stem cells was performed by dual SMAD inhibition under both adherent and free-floating EB culture conditions. After 10 days, we transferred cells to low-attachment culture plates and proliferated them as free-floating neurospheres. RNA was collected, transcribed to cDNA and analyzed for sonic hedgehog expression that plays an important role during proliferation process. NSPHs were analyzed by immunofluorescence imaging directly and upon continued differentiation. The EB-based approach yielded in higher numbers of cells expressing the neural stem cell marker Nestin, and showed in contrast to the adherent induction protocol increased expression levels of sonic hedgehog. Although improvements to culture consistency and reliability are desirable, the EB-based protocol appears to be superior to the adherent protocol for both, the proliferation and differentiation capacity.

## Introduction

Deriving and cultivating human neurons *in vitro* has always posed a particular challenge due to the need of proliferating early precursors. Different approaches to derive and cultivate human neural precursors and differentiating them into mature neurons have been established over time: (i) Primary neurons from aborted fetuses are ethically controversial and technically problematic due to their low yield. (ii) Human embryonic stem cells were isolated from *in-vitro-*fertilized embryos in the cleavage stage (Thomson et al., 1998). While this approach still has similar ethical problems as the use of primary neurons from fetuses, the cell yield can be increased through the step-wise process of neural induction to neural stem cells, proliferation, and differentiation to mature neurons. (iii) In 2007, the field of stem cell research was revolutionized by the development of induced pluripotent stem cells (iPSCs) (Takahashi et al., 2007). Since iPSCs are generated from adult human fibroblasts, ethical concerns are avoided and it is possible to generate patient-specific neurons. In many subsequent studies, the neural induction of pluripotent stem cells to neural stem cells (NSCs) was directly followed by differentiation to mature neurons (e.g., Dimos et al., 2008; Karumbayaram et al., 2009; Hartfield et al., 2014).

However, it is desirable to establish proliferating NSC cultures to improve their consistency by omitting repeated induction steps and to simplify the experimental procedures. In the past decade, different protocols have been published for the generation of proliferating NSC cultures from pluripotent cells. Most protocols relied on a combination of free-floating aggregates called embryoid bodies (EB) followed by adherent culture conditions (Zhang et al., 2001; Koch et al., 2009). More recent protocols used small molecule inhibitors of the activin/nodal and bone morphogenetic protein (BMP) pathway (also termed dual SMAD inhibition) (Kim et al., 2010) to enhance neural induction (Reinhardt et al., 2013). Some protocols reached the limits of expandability (Elkabetz et al., 2008; Reinhardt et al., 2013) or were not able to generate neurons of the peripheral nervous system (Li et al., 2011). One key study (Crompton et al., 2013) demonstrated the importance of intrinsic sonic hedgehog (SHH) signaling for a sufficient neuronal yield. SHH plays an important role in the proliferation process of NSCs, especially in the development from the NSC stage of radial glia cells to intermediate progenitor cells (Komada, 2012). Other protocols showed the possibility of neural induction under fully adherent conditions which resulted in a more uniform and consistent yield by minimizing the generation of non-neural cells. However, these protocols have not yet been employed to generate long-term proliferative cultures (Chambers et al., 2009).

While most protocols to derive NSCs from ESCs and iPSCs employed adherent cultivation, a more classical approach is cultivation as free-floating aggregates termed neurospheres (NSPHs). This culture type has mainly been employed for NSCs derived from embryonal or postnatal tissue in the past. It is considered a more natural environment for the cells due to its three-dimensional structure (Deleyrolle and Reynolds, 2009). It has been demonstrated that, at least for short-term expansion, combining the adherent induction protocol with NSPH cultivation is a feasible approach (Zhou et al., 2015). We aimed to answer the question how the difference between adherent and EB-based neural induction affects proliferation and differentiation to mature neurons, and which protocol holds more promise in combination with NSPH cultivation. Furthermore, we monitored the expression of sonic hedgehog (SHH) over time as an important factor for maintaining proliferativity.

## Materials and methods

### Neural induction and proliferation

Primary human dermal fibroblasts were reprogrammed to induced pluripotent stem cells (iPSCs) as described previously (Seibler et al., 2011). In short, fibroblasts were transduced with retroviral vectors for overexpression of OCT4, SOX2, cMYC, and KLF4. Four iPSC lines from healthy adult donors (who had given informed consent according to the ethical regulations of the University of Lübeck) were employed for the experiments. IPSC colonies were picked and cultured on irradiated mouse embryonic fibroblasts (MEF) in iPSC medium. The IPSCs were dissociated with accutase (Gibco, Carlsbad, CA, USA) for 15 min. Four hundred thousand cells were either transferred to ultra-low attachment 6-well culture plates (Corning, Corning, NY, USA) for the embryoid body (EB)-based protocol (Crompton et al., 2013), or plated on 6-well culture plates coated with matrigel (Corning, Corning, NY, USA) for the adherent induction (Chambers et al., 2009).

For adherent induction, cells were cultured in MEF-conditioned iPSC medium (CM) (see Supplemental Table 1) with FGF-2 (5 ng/ml; Merck Millipore, Darmstadt, Germany) and the ROCK-inhibitor Y-27632 (10 μM; Stemcell Technologies, Vancouver, Canada) for increased cell survival (Watanabe et al., 2007) until reaching confluency. For the EB-based protocol, cells were cultured in knockout serum replacement medium (KSR) (see Supplemental Table 1) with the ROCK-inhibitor Y-27632. From this point onwards, both protocols were identical except that cells were either cultured adherently or as EBs.

Neural induction was initiated in KSR by withdrawal of FGF-2. Dual SMAD inhibition was achieved by adding the small molecules SB-431542 (10 μM; Tocris, Bristol, UK) as a TGF-β inhibitor and LDN-193189 (100 nM; Stemgent, Cambridge, MA, USA) as a BMP-inhibitor. Successively, SB-431542, Y-27632, and LDN-193189 were withdrawn (at day 5, 7, and 10) and increasing amounts of N2B27 medium (25, 50, 75%) were added to the KSR medium. On day 10, cells from both induction protocols were dissociated with accutase for 15 min, transferred to ultra-low attachment 6-well culture plates. Single cells proliferated to NSPHs. NSCs were cultured in N2B27 medium (see Supplemental Table 1) supplemented with FGF-2 (10 ng/ml), EGF (epidermal growth factor, 20 ng/ml; Peprotech, Hamburg, Germany) and heparin (2 μg/ml; Merck Millipore, Darmstadt, Germany) and passaged when the NSPHs grew to a sufficient size so that a central dark core became apparent. The medium was replaced twice per week. All four iPSC lines were induced by the adherent induction protocol resulting in 10 individual experiments and two iPSC lines were induced by the EB-based induction protocol resulting in 4 individual experiments. At passages 2 and 5, cells were plated for immunostaining. Cells were dissociated by accutase treatment for 30 min and plated on poly-d-lysine (Sigma-Aldrich, St. Louis, MO, USA) and laminin (Roche, Basel, Switzerland)-coated coverslips (Hecht, Sondheim, Germany) at a density of 50,000 cells/well (~ 28,000 cells/cm^2^). We cultured the cells in N2B27 medium with FGF-2, EGF and heparin as described above. Cells were processed for immunostaining after reaching a sufficient number for analysis (usually after ~4 days).

### Differentiation

At passages 2 and 5, cells were dissociated to single cells and plated for differentiation as described above. We chose this approach in contrast to direct differentiation of NSPHs for a more standardized differentiation and better quantification by microscopy. When confluent, differentiation was initiated by withdrawal of FGF-2, EGF and heparin. To enhance neuronal differentiation, we added BDNF (brain-derived neurotrophic factor, 20 ng/ml), GDNF (glial cell-derived neurotrophic factor, 10 ng/ml), IGF1 (insulin-like growth factor 1, 10 ng/ml) (all from Peprotech, Hamburg, Germany), dibutyryl-cAMP (500 μM; EnzoLife Sciences, Lörrach, Germany) and the notch inhibitor DAPT (10 μM; Tocris, Bristol UK) for 30 days.

### Immunostaining

Cells were washed with PBS (Gibco, Carlsbad, CA, USA) and then fixed with 4% paraformaldehyde (Sigma-Aldrich, St. Louis, MO, USA) for 30 min at room temperature. After repeated washing with PBS, nonspecific binding sites were blocked for 45 min with PBS containing 5% normal donkey (Sigma-Aldrich, St. Louis, MO, USA) or goat serum (Invitrogen, Carlsbad, CA, USA), 0.1% Triton X-100 (AppliChem, Darmstadt, Germany) and 0.01% NaN_3_ (Sigma-Aldrich, St. Louis, MO, USA). Primary antibodies were incubated over night at 4 °C, followed by a washing step (3 × 15 min of PBS with 0.1% Triton X-100). Secondary antibodies were incubated for 2 h at room temperature. Both primary and secondary antibodies were diluted in PBS with 1% normal donkey or goat serum/ 0.1% Triton X-100 and 0.01% NaN_3_. For a list of the employed antibodies, see Supplemental Table 3. After a subsequent washing step, cover slips were mounted with media containing 4′, 6-Diamidin-2-phenylindol (DAPI) on cover slips and stored at 4°C. Slides were analyzed with a LSM 710 confocal microscope (Zeiss, Jena, Germany). Six random high-power fields were acquired and immunostained cells counted manually using ImageJ (NIH, Bethesda, MD, USA). The means of cell counts or percentages were calculated, along with the standard error of mean (SEM). An unpaired student *t*-test was performed to detect significant difference between both protocols with an alpha-level of 0.05. Multiple testing was corrected by the Holm-Sidak method. All statistical calculations were performed with GraphPad Prism 6 (La Jolla, CA, USA).

### Analysis of gene expression

The four iPSC lines were neurally induced under adherent or EB conditions and cultured until passage 8 as described above. After each passage, we extracted RNA from a third of the passaged cells (~500,000) cultured in ultra-low attachment 6-well culture plates (QIAshredder kit, RNeasy Mini Kit, Qiagen, Hilden, Germany). Subsequently, RNA was transcribed to cDNA by the Maxima First Strand cDNA Synthesis Kit for RT-qPCR (Thermo Fisher Scientific, Waltham, MA, USA).

We analyzed sonic hedgehog (SHH) expression by regular RT-PCR following the standard protocol provided (MP Biomedicals, Eschwege, Germany) (for the primer sequence see Supplemental Table 2). The PCR product was analyzed by agarose gel electrophoresis with β-actin as control. Percentages of positive cell lines were compared by Fisher's exact test.

## Results

### Cultivation, induction, and differentiation of cells

**Figure 1:**
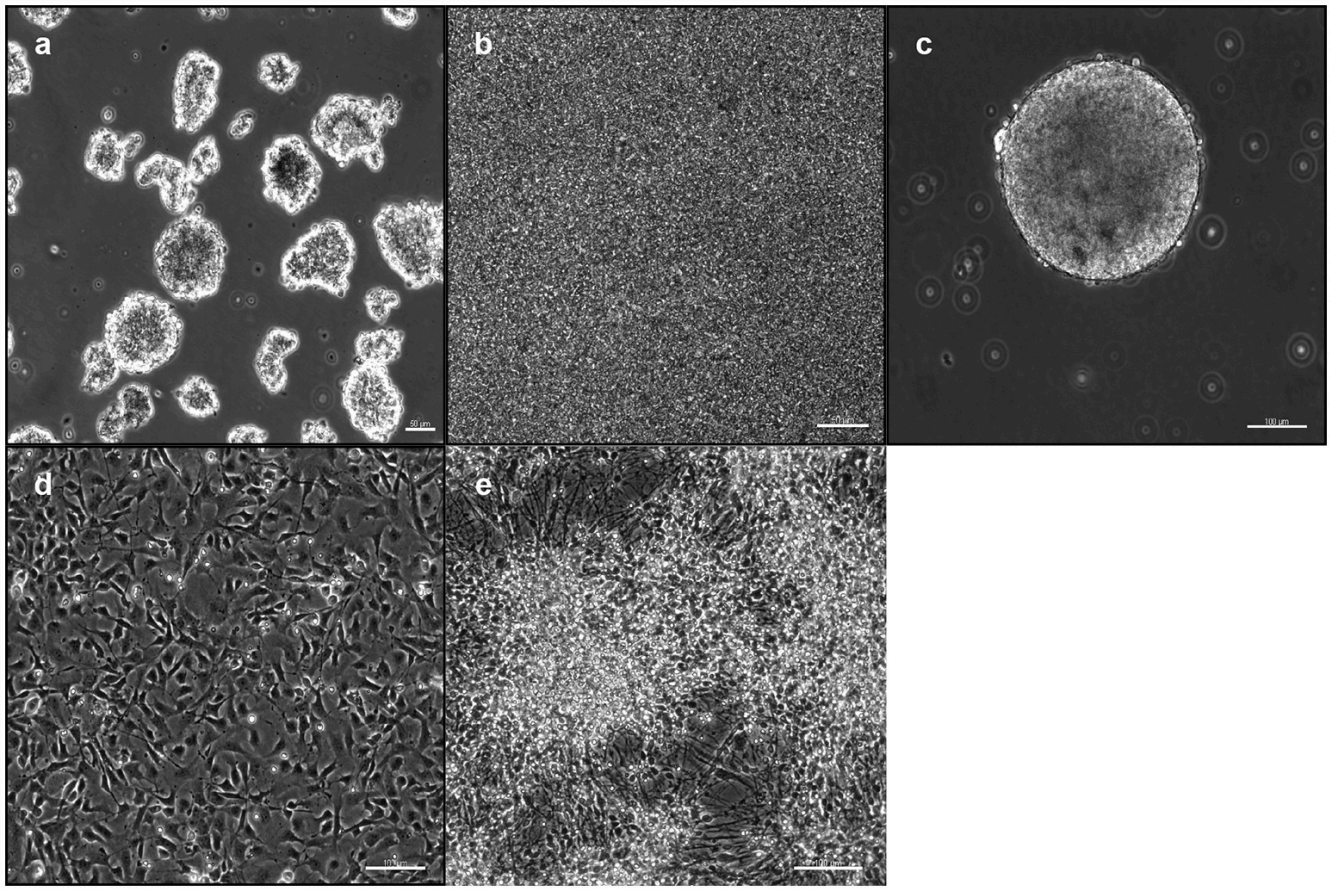
Induction and cultivation of NSCs from iPSCs: IPSCs were either plated as free-floating EBs **(a)** or plated and proliferated to high density **(b)** for neural induction. After 10 days, cells were dissociated and replated under low-adherent conditions for the formation of NSPHs **(c)**. For immunofluorescence analysis, cells were replated under adherent conditions **(d)** and differentiated for 30 days **(e)**. EB, embryoid body; iPSC, induced pluripotent stem cell; NPSH, neurosphere; NSC, neural stem cell. Scale bars: **(a)** 50 μm, **(b)** 50 μm, **(c)** 100 μm, **(d)** 100 μm, **(e)** 100 μm.

**Figure 2:**
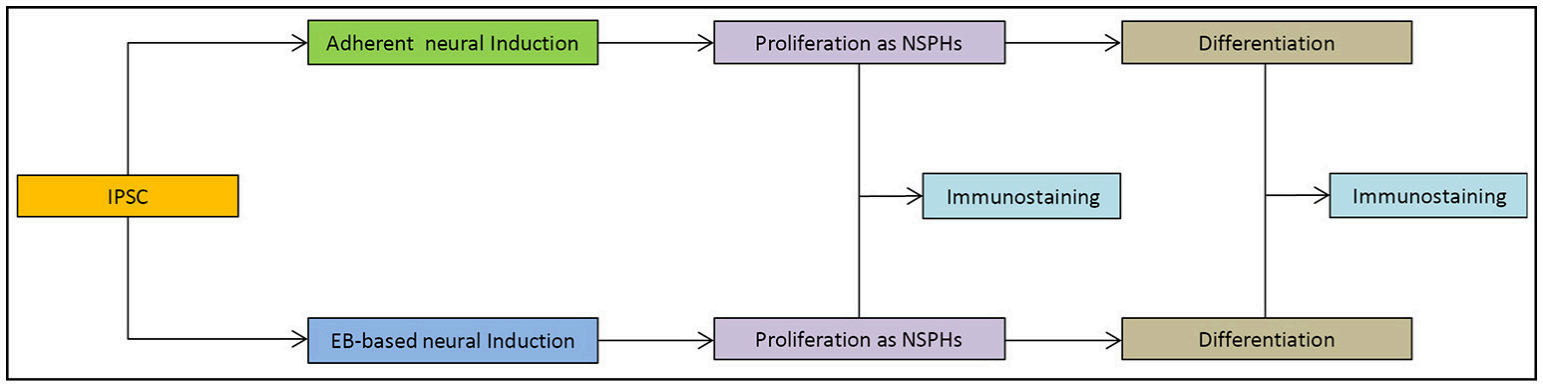
Schematic overview of experiments: outline of the study.

IPSCs reprogrammed from primary human dermal fibroblasts were dissociated to single cells. Subsequently they were either transferred to ultra-low attachment 6-well culture plates for the EB-based protocol (Figure 1a) or plated on 6-well culture plates for the adherent induction (Figure 1b). Neural induction was initiated and performed by dual SMAD inhibition for both protocols. Cells proliferated to NSPHs when transferred to ultra-low attachment culture plates on day 10. NSCs were passaged when NSPHs grew to a sufficient size so that a central dark core became apparent (Figure 1c). These occurred in average after 20.7 ± 1.38 days (with considerably shorter culture times at the beginning). Following dissociation, cells proliferated to new NSPHs. Both protocols shared the disadvantage that for all lines proliferation slowed down and finally stopped before passage 10 (at ~200 days). Thus, it was only possible to obtain a sufficient number of cells for analysis by immunofluorescence at passages 2 and 5. Cells were plated for immunostaining and differentiation at those time points. After reaching a sufficient number (Figure 1d), cells were directly processed for analysis or differentiation was initiated. In the latter case, cells were processed for analysis following 30 days of differentiation (Figure 1e). For a schematic overview, see Figure 2.

### Immunophenotype of NSCs and differentiated cells

#### NSCs under proliferative conditions

**Figure 3:**
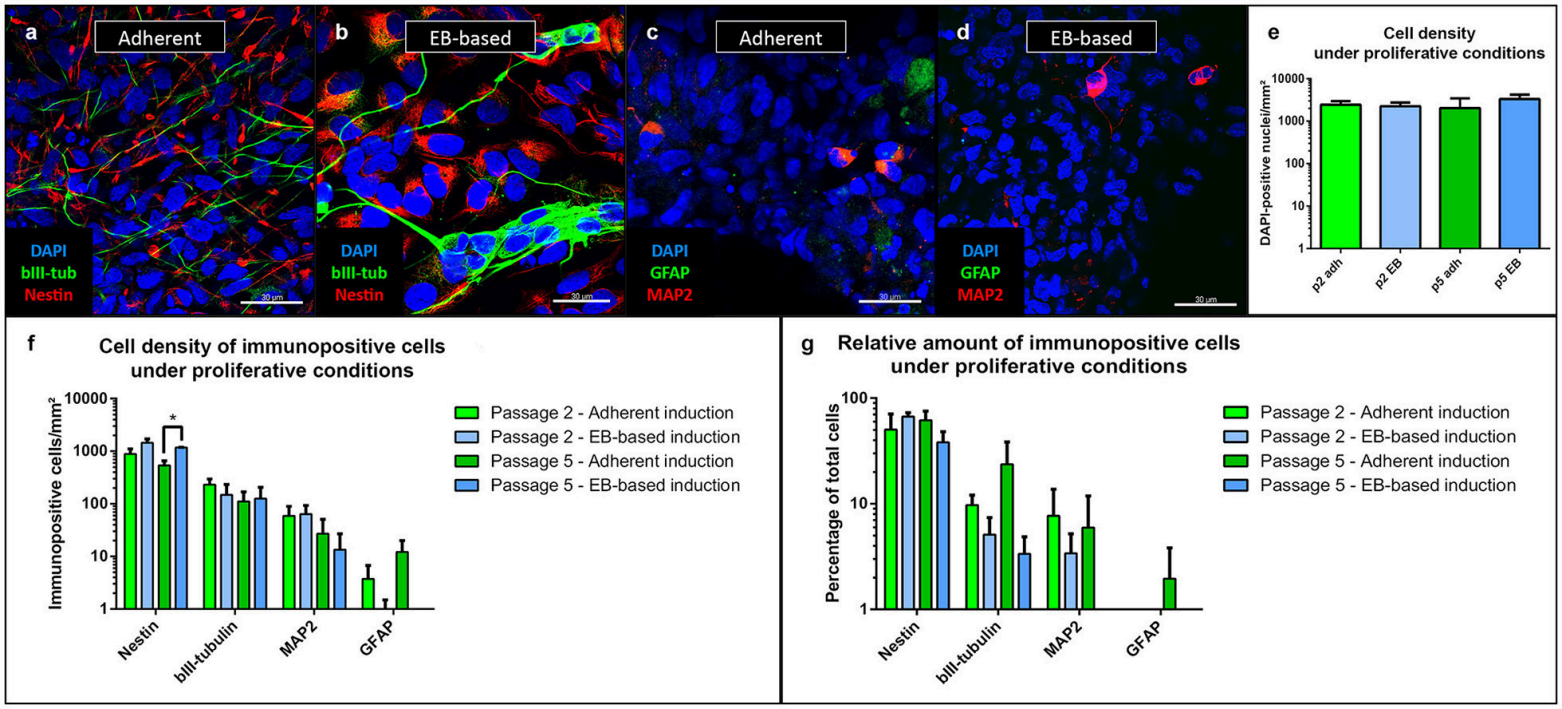
Immunofluorescence analysis of NSCs under proliferative conditions. Irrespective of the induction protocol chosen, the majority of cells expressed the intermediate filament Nestin (associated with NSCs); fewer cells expressed the early neuronal β-III-tubulin **(a,b)**. MAP2, a marker of mature neurons and expressed in only few cells, showed a low rate of spontaneous differentiation **(c,d)**. GFAP-positive astrocytes were only present in low numbers after adherent and absent after EB-based induction **(c,d)**. Density of cells did not differ (as intended as cells proliferated until near confluency before analysis) **(e)**. Density of Nestin-positive cells were significantly different between the adherent and EB-based protocol at passage 5 (*p* = 0.002, adherent: *n* = 10 EB: *n* = 4). There were no other significant differences between the induction protocols during cultivation for β-III-tubulin, MAP2 and GFAP **(f)**. The relative numbers of Nestin-, β-III-tubulin-, MAP2-, and GFAP-positive cells demonstrated no significant differences between the induction protocols **(g)**. All statistical analyses: unpaired student-*t*-test, correction for multiple testing by the Holm-Sidak method, error bars correspond to the standard error of the mean. EB, embryoid body; GFAP, glial fibrillary acidic protein; MAP2, microtuble-associated protein 2; NSC, neural stem cell. Scale bars: **(a)** 30 μm, **(b)** 30 μm, **(c)** 30 μm, **(d)** 30 μm. ^*^Significant with *p*-value < 0.05.

During the entire cultivation period, the majority of cells under proliferative conditions expressed the neural stem cell marker Nestin (Figures 3a,b). Additionally, we stained the cells with MAP2 as a marker for mature neurons and a sign of spontaneous differentiation (Figures 3c,d). The density of cells was similar in the experiments regardless of protocol or passage number (Figure 3e). The EB-based protocol yielded a significantly higher density of Nestin-positive cells at passage 5 (*p* = 0.002, *n* = 8, Figure 3f). However, the relative number of Nestin-positive cells did not show any significant difference between the different protocols and passages (Figure 3g). In comparison to the number of Nestin-positive cells, fewer cells expressed the neuronal progenitor marker β-III-tubulin (Katsetos et al., 2003) with similar numbers for all inducing conditions and passages (Figures 3a,b,f).

Relative numbers of β-III-tubulin-positive cells showed a trend toward higher values for the adherent induction protocol without reaching significant levels (Figure 3g). Only a low number of cells expressed MAP2 with similar amounts for both protocols and passages (Figure 3f). Relative numbers of MAP2-positive cells showed a trend to lower values for the EB-based protocol without reaching significant levels (Figure 3g). GFAP-positive cells were almost absent for the adherent induction protocol (Figure 3c). Proliferating cultures after EB-based induction were void of GFAP-positive cells (Figure 3d). Relative numbers of GFAP-positive cells showed a trend to lower values for the EB-based protocol without reaching significant levels (Figure 3g). For statistical values, see Supplemental Table 4.

#### Cellular identity after differentiation

**Figure 4:**
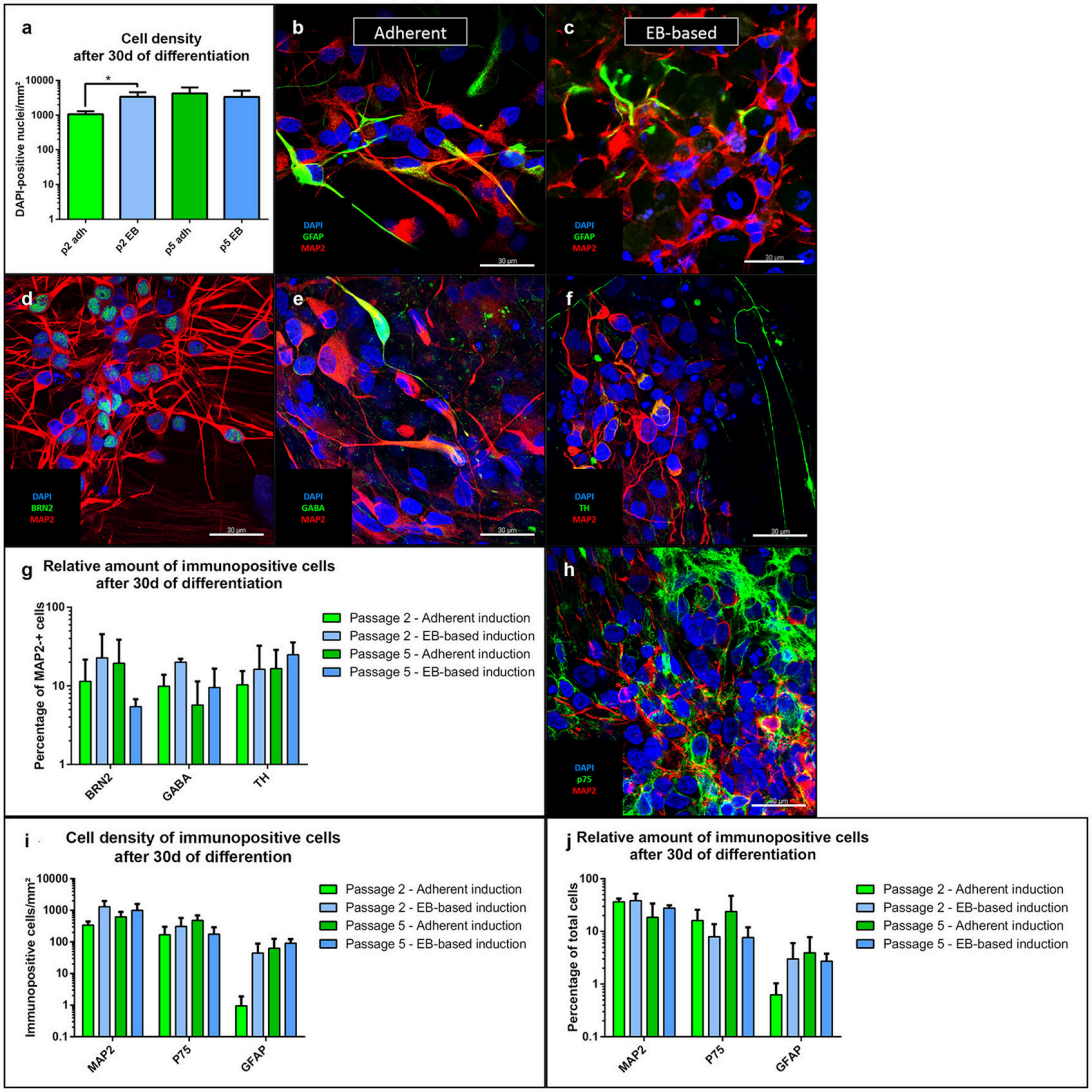
Immunofluorescence analysis of NSCs after 30 days of differentiation. The total cell number after differentiation was significantly higher for the EB-based protocol after passage 2 (*p* = 0.01, adherent: *n* = 10 EB: *n* = 4), but not after passage 5 **(a)**. NSCs from both induction protocols differentiated into MAP2-positive neurons and GFAP-positive astrocytes **(b,c)**. MAP2-positive cells co-expressed the cortical marker BRN2, GABA, and TH **(d–f)**. There were no statistically significant differences between induction protocols and passages for the distinct neuronal markers with a considerably high variability **(i)**. MAP2-positive cells showed a trend to higher absolute numbers for the EB-based protocol **(i)**. The relative numbers of MAP2-positive cells showed only a trend to higher proportions for the EB-based protocol **(j)**. GFAP-positive cells were only present at low absolute and relative numbers with no statistically significant differences **(i,j)**. P75-positive neural crest cells were also present under all inducing conditions and at all-time points **(h)**. No statistically significant differences for absolute and relative amounts were present. However, the adherent induction protocol showed a trend to higher relative amounts of p75-positive cells **(i,j)**. All statistical analyses: unpaired student-*t*-test, correction for multiple testing by the Holm-Sidak method, error bars correspond to the standard error of the mean. EB, embryoid body; GABA, γ-aminobutyric acid; GFAP, glial fibrillary acidic protein; MAP2, microtuble-associated protein 2; NSC, neural stem cell; TH, tyrosine hydroxylase. Scale bars: **(b)** 30 μm, **(c)** 30 μm, **(d)** 30 μm. ^*^Significant with *p*-value < 0.05.

For differentiation after passage 2, the total cell number of the EB-based induction protocol was significantly higher compared to the adherent protocol (*p* = 0.01, *n* = 8). However, after passage 5, total cell numbers were similar (Figure 4a). Cells differentiated into mature MAP2-positive neurons (Figures 4b,c) with both induction protocols. There was nominal significance for a higher number of neurons for the EB-based protocol after passage 2 and a trend after passage 5 compared to the adherent induction (Figure 4i). Relative amounts of MAP2-positive cells showed a trend toward higher numbers for the EB-based protocol without reaching statistical significance (Figure 4j). For further characterization, we analyzed distinct neuronal subtypes. MAP2-positive cells showed no significant differences at any time point (with considerable variability between different lines) when comparing both protocols regarding the co-expression of the following neuronal markers (Figure 4g): BRN2 (a marker for cortical neurons, Dominguez et al., 2013, Figure 4d), GABA (Figure 4e), TH (expressed by dopaminergic neurons, Figure 4f). GFAP-positive cells were present in low numbers after differentiation (Figures 4b,c). There was a trend toward higher density after passage 2 for the EB-based neural induction (Figure 4i). Relative numbers showed no differences between protocols and passages (Figure 4j). As a marker for neural crest cells, we stained cells for p75 after differentiation (Figure 4h). There were no clear differences in density for p75-positiv cells (Figure 4i). However, there was a trend toward a higher relative amount for the adherent induction protocol (Figure 4j). For statistical values, see Supplemental Table 4.

### Expression of sonic hedgehog

**Figure 5:**
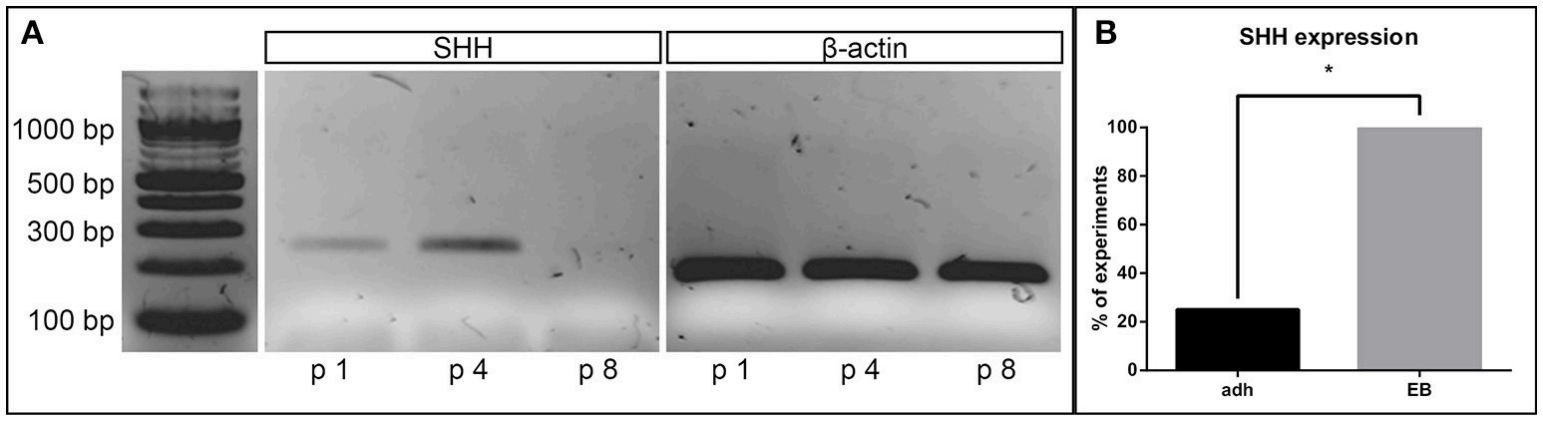
Sonic hedgehog expression. Representative gel electrophoresis of a RT-PCR on cDNA derived from a NSC line (EB-based neural induction) for SHH at different passages demonstrates absent expression after passage 8 (beta-actin as loading control) **(A)**. One hundred percentage of all lines induced by the EB-based protocol, but only 25% of the adherently induced ones expressed SHH at any time point during cultivation (*p* = 0.02, adherent: *n* = 10 EB: *n* = 4, Fisher's exact test) **(B)**. ^*^Significant with *p*-value < 0.05.

In its role as an important proliferative factor of NSCs, we analyzed the capacity of the cell lines to express SHH (Agathocleous et al., 2007), by evaluating the presence of the corresponding cDNA. All cell cultures derived from the EB-based protocol expressed SHH until passage 6. In higher passages, shortly before the cell lines stopped proliferating, the expression of SHH faded (Figure 5A). Of the 4 cell lines induced neurally with the adherent protocol, only one showed SHH expression at any passage. Thus, the number of cell lines expressing SHH were significantly higher for the EB-based protocol (*p* = 0.02, *n* = 8; Figure 5B).

## Discussion

The stable expansion of neural cell lines derived from pluripotent stem cells remains a demanding task: Pluripotent stem cells need to undergo neural induction and be transferred to proliferative culture conditions. Hereafter, immature, yet neurally committed neural precursors must be cultivated without spontaneous differentiation or loss of proliferativity.

For neural induction, besides the more classical EB-based protocol, a fully adherent protocol (Chambers et al., 2009) has been developed and successfully employed for directed differentiation toward mature neurons (Kriks et al., 2011). The advantages of this approach are considered gold standard over inducing conditions, as cell number and size cannot be easily controlled for EBs.

Cultivation of NSCs is also possible under adherent culture conditions, as well as three-dimensionally as free-floating NSPHs. As for neural induction, adherent culturing has been considered superior in terms of standardization and reproducibility (D'Aiuto et al., 2014). However, as the developing central nervous system represents a three-dimensional structure with regionally specified expression of transcription factors and neurotrophins, NSPHs are considered a more “natural” way of cultivating neural stem cells (Deleyrolle and Reynolds, 2009). Indeed, most protocols for cultivating primary NSCs from fetal tissue relied on the long-term cultivation of NSPHs (Pacey et al., 2006), whereas protocols deriving neural precursors from iPSCs or ESCs are mostly based on adherent culture (Koch et al., 2009; Falk et al., 2012; Reinhardt et al., 2013). Recent publications introduced free-floating culturing steps during neural induction and differentiation of iPSCs (Crompton et al., 2013; D'Aiuto et al., 2014; Zhou et al., 2015). The added value of NPSH may arise from the ability of creating their own favorable microenvironment, e.g., by the autonomous production of the proneural signal molecule SHH (Crompton et al., 2013).

Our aim was to compare adherent and EB-based neural induction protocols with respect to their influence on maintained proliferativity, neural identity, and differentiation.

Furthermore, we aimed to evaluate the combination of both induction protocols with the free-floating proliferation step, which has been used in the past for proliferating primary neural stem cells. The key readout of the analysis was immunostainings of proliferating NSCs and differentiated neurons. Additionally, we analyzed the expression of SHH as an important factor for sustained neural proliferatitvity.

The culture conditions during neural induction had a significant effect on certain aspects of the performance of the NSC lines during proliferation and differentiation. The cell lines induced via EBs contained a higher density of Nestin-positive NSCs (significantly at passage 5) and after differentiation resulted in a higher density of total cells and a trend toward more MAP2-positive neurons (nominally significant at passage 2). Relative numbers of MAP2-positive neurons after differentiation showed a trend toward higher values for the EB-based protocol. Crompton et al. showed that neural cultures induced from iPSCs via EBs have an intrinsic capacity to produce SHH (Crompton et al., 2013). Our results were in line with these findings, although we haven't assessed the production of SHH *per se*, but evaluated the presence of SHH mRNA. In every cell line induced by the EB-based protocol SHH mRNA was present at least during early passages. For the NSCs from the adherent protocol this was true only for some of the cell lines. It is therefore possible that the superior performance associated with the EB-based neural induction was due to the induction method.

Both protocols yielded similar proportions of GABAergic, dopaminergic, and cortical neurons. However, the relative amounts of the different subtypes showed considerable variability between the different lines. The proliferation as NSPHs being a three-dimensional system is considered a good approximation to *in-vivo* neurogenesis and a suitable *in-vitro* model for NSCs (Marshall et al., 2007). However, NSPHs are also very sensitive to changes in culture conditions (Jensen and Parmar, 2006), a possible reason for the high variability.

Taking into consideration the temporal connection between the cessation of SHH mRNA expression and the reduction of proliferation, the role of SHH in the proliferation process could be of importance. In this regard, the EB-based protocol has a advantage over the adherent protocol in terms of higher numbers of Nestin-positive NSCs and neurons after differentiation. However, it is obvious that other factors must play an important role as well for sustained proliferation, as NSC lines induced by the adherent protocol proliferate despite of absent endogenous SHH mRNA expression. Further experiments including overexpression of SHH or extrinsic addition of SHH-agonists may improve the capability of proliferation for a longer period. A more recent protocol for long-term cultivation of neural precursors from human iPSCs indeed already successfully employed the SHH-agonist purmorphamine for this purpose (Reinhardt et al., 2013). However, Reinhardt et al. employed a complex cocktail of small molecules to achieve long-term proliferation in comparison to the more basic protocol we used.

In conclusion, we confirmed the dual SMAD inhibition paradigm as a reliable method for neural induction. It is effective and, by using small molecules, cost-efficient and simple to apply. Furthermore, it is successful under adherent as well as free-floating conditions. However, in direct comparison, the EB-based neural induction emerged as the more promising protocol. Although differences were rather subtle, a higher proliferation and differentiation capacity as well as the presence of SHH mRNA at least during early passages suggests that this method of neural induction may be the preferred one during early cultivation. However, our data also imply that NSPHs, despite their long-standing and successful role in the cultivation of primary stem cells, may not be a feasible option for iPSC-derived neural lines. There are several options for further investigation and improvement of neural induction and proliferation protocols. For example, it would be of interest to modify the culture conditions, such as cultivation under physiological hypoxia (Bilican et al., 2014), combine the proliferation with patterning signals or to try to improve the duration of proliferation by overexpressing SHH. Furthermore, a direct comparison of adherent cultivation with NSPH cultivation subsequent to EB-based neural induction may conclusively clarify the question, which one of the cultivation methods is indeed superior.

## Author contributions

MP: Concept and design, data acquisition, analysis and interpretation, manuscript writing. VK: Data acquisition, analysis and interpretation. FS: Data acquisition, analysis and interpretation. PS: Concept and design, data analysis and interpretation (iPSC). CK: Concept and design, manuscript writing, PC: Concept and design, data acquisition, analysis and interpretation, manuscript writing.

### Conflict of interest statement

The authors declare that the research was conducted in the absence of any commercial or financial relationships that could be construed as a potential conflict of interest.

## Abbreviations

BMP: bone morphogenetic protein
EB: embryoid bodies
EGF: epidermal growth factor
FGF: fibroblast growth factor
GFAP: glial fibrillary acidic protein
iPSC: induced pluripotent stem cells.
KSR: knockout serum replacement medium
MAP: microtubule associated protein
MEF: mouse embryonic fibroblasts
NSPH: neurospheres
NSC: neural stem cells
SHH: sonic hedgehog.
