# Supplementary material for: Adherent vs. Free-Floating Neural Induction by Dual SMAD Inhibition for Neurosphere Cultures Derived from Human Induced Pluripotent Stem Cells
Source: Front Cell Dev Biol. 2018 Feb 6;6:3. doi: 10.3389/fcell.2018.00003 (PMC5807902; doi:10.3389/fcell.2018.00003)
Supplement: Supplementary file 1 [file DataSheet1.DOCX]

Supplementary Material

Adherent versus free-floating neural induction by dual SMAD inhibition for neurosphere cultures derived from human induced pluripotent stem cells

Martje Gesine Pauly, Victor Krajka, Felix Stengel, Philip Seibler, Christine Klein, Philipp Capetian*

*** Correspondence:** Dr. med. Philipp Capetian Philipp.Capetian@neuro.uni-luebeck.de

# Composition of culture medium

## Medium conditioned by mouse embryonic fibroblasts (CM)

| KnockOut™ DMEM/F-12 (1X) | 400 ml | Gibco, Carlsbad, US |
| --- | --- | --- |
| KnockOut™ Serum Replacement | 100 ml | Gibco, Carlsbad, US |
| HEPES buffer 1M | 7,5 ml | Gibco, Carlsbad, US |
| L-Glutamin | 5 ml | Gibco, Carlsbad, US |
| MEM NEAA 100x | 5 ml | Gibco, Carlsbad, US |
| 2-Mercaptoethanol 50 mM | 1 ml | Gibco, Carlsbad, US |

## Knock-out serum replacement (KSR) medium

| KnockOut™ DMEM/F-12 (1X) | 425 ml | Gibco, Carlsbad, US |
| --- | --- | --- |
| KnockOut™ Serum Replacement | 75 ml | Gibco, Carlsbad, US |
| L-Glutamin | 5 ml | Gibco, Carlsbad, US |
| MEM NEAA 100x | 5 ml | Gibco, Carlsbad, US |
| Pen Strep | 5 ml | Gibco, Carlsbad, US |
| 2-Mercaptoethanol 50 mM | 1 ml | Gibco, Carlsbad, US |
| Fungizone | 500 µl | Invitrogen, Carlsbad, US |

## N2B27 medium

| Neurobasal® Medium (1X) | 250 ml | Life Technologies, Carslbad, US |
| --- | --- | --- |
| KnockOut™ DMEM/F-12 (1X) | 250 ml | Gibco, Carlsbad, US |
| HEPES buffer 1M | 5 ml | Gibco, Carlsbad, US |
| N2-Supplement (100X) | 5 ml | Gibco, Carlsbad, US |
| NeuroCult™ SM1 Neuronal Supplement | 10 ml | Stemcell Technologies, Vancouver, CA |
| Pen Strep | 5 ml | Gibco, Carlsbad, US |
| MEM NEAA 100x | 5 ml | Gibco, Carlsbad, US |
| Fungizone | 500 µl | Invitrogen, Carlsbad, US |

# List of primer sequences

| **Gene** | **Forward primer** | **Reverse primer** |
| --- | --- | --- |
| DACH1 | GGR GRG CAA TGT GGA ACA AG | CTT AGG AGG CCT TCC AGG TC |
| EMX2 | ACC TTC TAC CCC TGG CTC AT | GCC CAC CAC GTA GTG ATT CT |
| FOXG1 | TGT TGA CTC AGA ACT CGC TGG | CTG CTC TGC GAA GTC ATT GAC |
| GSX2 | TTA AGG GCC AGT TCT CTT CG | CTC CGG AGT CGA GAC AGG TA |
| KROX20 | CCA AGG CCG TAG ACA AAA TC | GGA TAT GGG AGA TCC AAC GA |
| MAP2 | CAG GTG GCG GAC GTG TGA AAA TTG AGA GTG | CAC GCT GGA TCT GCC TGG GGA CTG TG |
| MASH1 | CAT CTC CCC CAA CTA CTC CA | AAC GCC ACT GAC AAG AAA GC |
| NESTIN | CGT TGG AAC AGA GGT TGG AG | TCT GGG GTC CTA GGG AAT TG |
| SHH | AGA AAC TCC GAG CGA TT | CCT CGT AGT GCA GAG ACT CC |
| β -Actin | TGA AGT GTG ACG TGG ACA TC | GGA GGA GCA ATG ATC TTG AT |

# List of antibodies

## Primary antibodies

| **Antigen** | **Isotype** | **Dilution** | **Manufacture** | **Catalogue number** |
| --- | --- | --- | --- | --- |
| GFAP | rabbit | 1:1000 | ZYTOMED Systems GmbH, Berlin, GE | RBK037-05 |
| MAP2 | mouse | 1:800 | Millipore, Billerica, US | MAB378 |
| NESTIN | rat | 1:1000 | abcam, Cambridge, GB | AB18102 |
| p75 | rabbit | 1:1000 | Promega, Madison, US | G3231 |
| β-III-tubulin | rabbit | 1:500 | Covance, Princeton, US | PRB-435P |

## Secondary antibodies

| **Antigen** | **Isotype** | **Dilution** | **Fluorophore** | **Manufacture** | **Catalogue number** |
| --- | --- | --- | --- | --- | --- |
| Rabbit IgG | donkey | 1:800 | Alexa Fluor® 568 | Life Technologies™, Carlsbad, US | A10042 |
| Rabbit IgG | goat | 1:1000 | Alexa Fluor® 488 | Life Technologies™, Carlsbad, US | A11008 |
| Mouse IgG | donkey | 1:800 | Alexa Fluor® 647 | Invitrogen, Carlsbad, US | A31571 |
| Mouse IgG | goat | 1:1000 | Alexa Fluor® 488 | Invitrogen, Carlsbad, US | A10680 |
| Mouse IgG | goat | 1:800 | Alexa Fluor® 568 | Life Technologies™, Carlsbad, US | A11004 |
| Mouse IgG | donkey | 1:800 | Alexa Fluor® 488 | Invitrogen, Carlsbad, US | A21202 |
| Rat IgG | goat | 1:800 | Alexa Fluor® 568 | Invitrogen, Carlsbad, US | A11077 |

# Statistical analyzes

## Density of cells under proliferative conditions (DAPI-positive nuclei /mm²) - unpaired t-test with alpha=5.000%:

| **Passage number** | **P value** | **P value summary** | **Mean ± SEM of Adherent** | **Mean ± SEM of EB** | **Difference between means** | **95% confidence interval** |
| --- | --- | --- | --- | --- | --- | --- |
| Passage 2 | 0,8118 | ns | 2447 ± 516,7 | 2239 ± 498,4 | -208,3 ± 853,8 | -2088 to 1671 |
| Passage 5 | 0,6134 | ns | 2040 ± 1403 | 3329 ± 898,6 | 1290 ± 2395 | -4868 to 7447 |

## Relative amount of immunopositive cells under proliferative conditions (% of total cells) - Statistical significance determined using the Holm-Sidak method, with alpha=5.000%:

| Passage 2 | |  |  |  |  |  |  |
| --- | --- | --- | --- | --- | --- | --- | --- |
|  | **P value** | **P value summary** | **Mean ± SEM of Adherent** | **Mean ± SEM of EB** | **Difference between means** | **SE of difference** | **t ratio** |
| Nestin | 0,60545 | ns | 50,36 ± 10,21 | 67 ,0 ± 5,87 | -16,64 | 31,291 | 0,531782 |
| bIII-tubulin | 0,270221 | ns | 9,7 ± 2,41 | 5,1 ± 2,32 | 4,6 | 3,96213 | 1,16099 |
| MAP2 | 0,652602 | ns | 7,7 ± 6,03 | 3,4 ± 1,83 | 4,3 | 9,2934 | 0,462694 |
| GFAP | 0,454823 | ns | 0,15 ± 0,10 | 0,03 ± 0,03 | 0,12 | 0,15489 | 0,774743 |
|  |  |  |  |  |  |  |  |
| Passage 5 | |  |  |  |  |  |  |
|  | **P value** | **P value summary** | **Mean ± SEM of Adherent** | **Mean ± SEM of EB** | **Difference between means** | **SE of difference** | **t ratio** |
| Nestin | 0,358902 | ns | 61,86 ± 13,64 | 38,2 ± 9,99 | 23,66 | 23,4287 | 1,00987 |
| bIII-tubulin | 0,4517 | ns | 23,67 ± 14,97 | 3,36 ± 1,52 | 20,31 | 24,8951 | 0,815823 |
| MAP2 | 0,592583 | ns | 5,95 ± 5,90 | 0,32 ± 0,32 | 5,64 | 9,87404 | 0,571195 |
| GFAP | 0,562499 | ns | 1,95 ± 1,88 | 0 ± 0 | 1,95 | 3,14584 | 0,619866 |

## Density of immunopositive cells under proliferative conditions (immunopositive cells /mm²) - Statistical significance determined using the Holm-Sidak method, with alpha=5.000%:

| Passage 2 | |  |  |  |  |  |  |
| --- | --- | --- | --- | --- | --- | --- | --- |
|  | **P value** | **P value summary** | **Mean ± SEM of Adherent** | **Mean ± SEM of EB** | **Difference between means** | **SE of difference** | **t ratio** |
| Nestin | 0,167015 | ns | 890 ± 218,2 | 1448 ± 275,4 | -558 | 377,105 | 1,4797 |
| bIII-tubulin | 0,489033 | ns | 231,6 ± 64,4 | 149 ± 84,7 | 82,6 | 115,727 | 0,713748 |
| MAP2 | 0,931854 | ns | 59,28 ± 30,2 | 63,81 ± 29,1 | -4,53 | 51,8758 | 0,087324 |
| GFAP | 0,551525 | ns | 3,73 ± 3,0 | 0,75 ± 0,75 | 2,99 | 4,88023 | 0,612676 |
|  |  |  |  |  |  |  |  |
| Passage 5 | |  |  |  |  |  |  |
|  | **P value** | **P value summary** | **Mean ± SEM of Adherent** | **Mean ± SEM of EB** | **Difference between means** | **SE of difference** | **t ratio** |
| Nestin | 0,002189 | * | 542,6 ± 122,8 | 1182 ± 10,46 | -642 | 136,377 | 4,70754 |
| bIII-tubulin | 0,885986 | ns | 111,2 ± 57,33 | 125,5 ± 80,7 | -14,3 | 96,169 | 0,148697 |
| MAP2 | 0,655434 | ns | 27,03 ± 23,53 | 13,45 ± 13,45 | 13,58 | 29,1477 | 0,465903 |
| GFAP | 0,397301 | ns | 12,2 ± 7,88 | 0 ± 0 | 12,2 | 13,1858 | 0,92524 |

## Density of cells after 30d of differentiation (DAPI-positive nuclei /mm²) - unpaired t-test with alpha=5.000%:

| **Passage number** | **P value** | **P value summary** | **Mean ± SEM of Adherent** | **Mean ± SEM of EB** | **Difference between means** | **95% confidence interval** |
| --- | --- | --- | --- | --- | --- | --- |
| Passage 2 | 0,0129 | * | 1060 ± 230,1 | 3398 ± 1172 | 2338 ± 755,6 | 628,3 to 4047 |
| Passage 5 | 0,7934 | ns | 4213 ± 2089 | 3359 ± 1720 | -854,3 ± 2986 | -10356 to 8647 |

## Relative amount of immunopositive cells after 30d of differentiation (% of total cells) - Statistical significance determined using the Holm-Sidak method, with alpha=5.000%:

| Passage 2 | |  |  |  |  |  |  |
| --- | --- | --- | --- | --- | --- | --- | --- |
|  | **P value** | **P value summary** | **Mean ± SEM of Adherent** | **Mean ± SEM of EB** | **Difference between means** | **SE of difference** | **t ratio** |
| MAP2 | 0,878022 | ns | 36,49 ± 5,5 | 38,39 ± 13,5 | -1,9 | 12,0672 | 0,157452 |
| P75 | 0,487626 | ns | 16,1 ± 9,7 | 7,9 ± 5,8 | 8,2 | 10,3905 | 0,789183 |
| GFAP | 0,121881 | ns | 0,09 ± 0,09 | 1,993 ± 1,99 | -1,903 | 1,11211 | 1,71117 |
|  |  |  |  |  |  |  |  |
| Passage 5 | |  |  |  |  |  |  |
|  | **P value** | **P value summary** | **Mean ± SEM of Adherent** | **Mean ± SEM of EB** | **Difference between means** | **SE of difference** | **t ratio** |
| MAP2 | 0,618837 | ns | 18,51 ± 15,18 | 27,62 ± 3,73 | -9,11 | 15,6244 | 0,583062 |
| P75 | 0,573634 | ns | 23,73 ± 23,73 | 7,645 ± 4,38 | 16,085 | 24,1299 | 0,6666 |
| GFAP | 0,162322 | ns | 0 ± 0 | 3,49 ± 2,53 | -3,485 | 1,88948 | 1,84443 |

## Relative amount of immunopositive cells after 30d of differentiation (% of MAP2-positive cells) - Statistical significance determined using the Holm-Sidak method, with alpha=5.000%:

| Passage 2 | |  |  |  |  |  |  |
| --- | --- | --- | --- | --- | --- | --- | --- |
|  | **P value** | **P value summary** | **Mean ± SEM of Adherent** | **Mean ± SEM of EB** | **Difference between means** | **SE of difference** | **t ratio** |
| BRN2 | 0,608699 | ns | 11,41 ± 10,16 | 22,68 ± 22,68 | -11,27 | 21,2499 | 0,530356 |
| GABA | 0,167032 | ns | 9,886 ± 3,94 | 20,02 ± 2,04 | -10,134 | 6,7415 | 1,50323 |
| TH | 0,647608 | ns | 10,35 ± 5,09 | 16,23 ± 16,23 | -5,88 | 12,4364 | 0,472806 |
|  |  |  |  |  |  |  |  |
| Passage 5 | |  |  |  |  |  |  |
|  | **P value** | **P value summary** | **Mean ± SEM of Adherent** | **Mean ± SEM of EB** | **Difference between means** | **SE of difference** | **t ratio** |
| BRN2 | 0,616856 | ns | 19,37 ± 13,37 | 5,45 ± 1,33 | 13,92 | 25,0262 | 0,556217 |
| GABA | 0,699884 | ns | 5,7 ± 5.7 | 9,53 ± 7,0 | -3,83 | 9,02497 | 0,424378 |
| TH | 0,669034 | ns | 16,57 ± 12,05 | 24,85 ± 10,86 | -8,28 | 17,5364 | 0,47216 |

## Density of immunopositive cells after 30d of differentiation (immunopositive cells /mm²) - Statistical significance determined using the Holm-Sidak method, with alpha=5.000%:

| Passage 2 | |  |  |  |  |  |  |
| --- | --- | --- | --- | --- | --- | --- | --- |
|  | **P value** | **P value summary** | **Mean ± SEM of Adherent** | **Mean ± SEM of EB** | **Difference between means** | **SE of difference** | **t ratio** |
| MAP2 | 0,0334572 | ns | 382,3 ± 106,8 | 1302 ± 666 | -961,3 | 390,167 | 2,46382 |
| P75 | 0,661051 | ns | 168 | 309,9 | -141,9 | 300,176 | 0,472722 |
| GFAP | 0,111084 | ns | 0,95 ± 0,95 | 44,5 ± 44,5 | -43,55 | 24,6506 | 1,76669 |
|  |  |  |  |  |  |  |  |
| Passage 5 | |  |  |  |  |  |  |
|  | **P value** | **P value summary** | **Mean ± SEM of Adherent** | **Mean ± SEM of EB** | **Difference between means** | **SE of difference** | **t ratio** |
| MAP2 | 0,53604 | ns | 619,4 ± 276,1 | 991,7 ± 600,2 | -372,3 | 560,638 | 0,664065 |
| P75 | 0,360921 | ns | 480,3 ± 209,2 | 174,8 ± 118,0 | 305,5 | 284,036 | 1,07557 |
| GFAP | 0,72623 | ns | 62,9 ± 62,9 | 91,2 ± 31,4 | -28,3 | 70,302 | 0,402549 |
